# Dynamic properties of surfactant-enhanced laser-induced vapor bubbles for lithotripsy applications

**DOI:** 10.1117/1.JBO.26.1.018001

**Published:** 2021-01-29

**Authors:** Nicholas C. Giglio, Thomas C. Hutchens, Austin A. South, Nathaniel M. Fried

**Affiliations:** a University of North Carolina at Charlotte, Department of Physics and Optical Science, Charlotte, North Carolina, United States; b University of North Carolina at Charlotte, Department of Mechanical Engineering, Charlotte, North Carolina, United States

**Keywords:** kidney stone disease, lithotripsy, surfactant, thulium fiber laser, vapor bubbles

## Abstract

**Significance:** Water is a primary absorber of infrared (IR) laser energy, and urinary stones are immersed in fluid in the urinary tract and irrigated with saline during IR laser lithotripsy. Laser-induced vapor bubbles, formed during lithotripsy, contribute to the stone ablation mechanism and stone retropulsion effects.

**Aim:** Introduction of a surfactant may enable manipulation of vapor bubble dimensions and duration, potentially for more efficient laser lithotripsy.

**Approach:** A surfactant with concentrations of 0%, 5%, and 10% was tested. A single pulse from a thulium fiber laser with wavelength of 1940 nm was delivered to the surfactant through a 200-μm-core optical fiber, using a wide range of laser parameters, including energies of 0.05 to 0.5 J and pulse durations of 250 to 2500  μs.

**Results:** Bubble length, width, and duration with surfactant increased on average by 29%, 17%, and 120%, compared with water only.

**Conclusions:** Our study demonstrated successful manipulation of laser-induced vapor bubble dimensions and duration using a biocompatible and commercially available surfactant. With further study, use of a surfactant may potentially improve the “popcorn” technique of laser lithotripsy within the confined space of the kidney, enable non-contact laser lithotripsy at longer working distances, and provide more efficient laser lithotripsy.

## 1 Introduction

Urinary stone disease affects about 10% of Americans.^1^ A successful minimally invasive method for treatment of small- to moderate-sized (<2  cm diameter) urinary stones utilizes an infrared (IR) laser, optical fiber, and flexible ureteroscope. The ureteroscope is introduced into the urinary tract, and then a flexible silica optical fiber is inserted through the working channel of the ureteroscope and placed in contact or in close proximity to the stone. The IR laser energy is then delivered in a long pulse mode, through an optical fiber, to break up the stone into sufficiently small fragments for either grasping and removal with a stone basket instrument or into smaller particles known as stone “dust” to be spontaneously passed through the patient’s urinary tract.

The holmium:YAG laser, with an IR wavelength of 2120 nm, has been the gold standard laser for lithotripsy for the past several decades because of (1) its ability to successfully treat a wide variety of different stone chemical compositions as well as soft urinary tissues from a single laser platform, (2) its proven safety record for use in endourology, and (3) the commercial availability of low-cost, biocompatible, small, flexible, and robust silica optical fibers for transmission and delivery of the laser energy within the urinary tract.^2^

More recently, a thulium fiber laser (TFL), with an IR wavelength of 1940 nm, has been developed and has been demonstrated both in the laboratory and in the clinic to be a feasible alternative to the holmium:YAG laser for lithotripsy.^3^^–^^7^ The TFL has several notable advantages, including: (1) an IR laser wavelength that more closely matches a major water absorption peak, translating into a four times lower stone ablation threshold and higher stone ablation rates, (2) use with smaller and more flexible optical fibers that do not inhibit maximum ureteroscope flexion and also use less space within the working channel of the ureteroscope, thus enabling higher irrigation rates for enhanced visibility and safety, and (3) a more compact, quieter, fan-cooled, high-power laser system operated from a standard 110- or 220-V electrical receptacle.

**Fig. 1:**
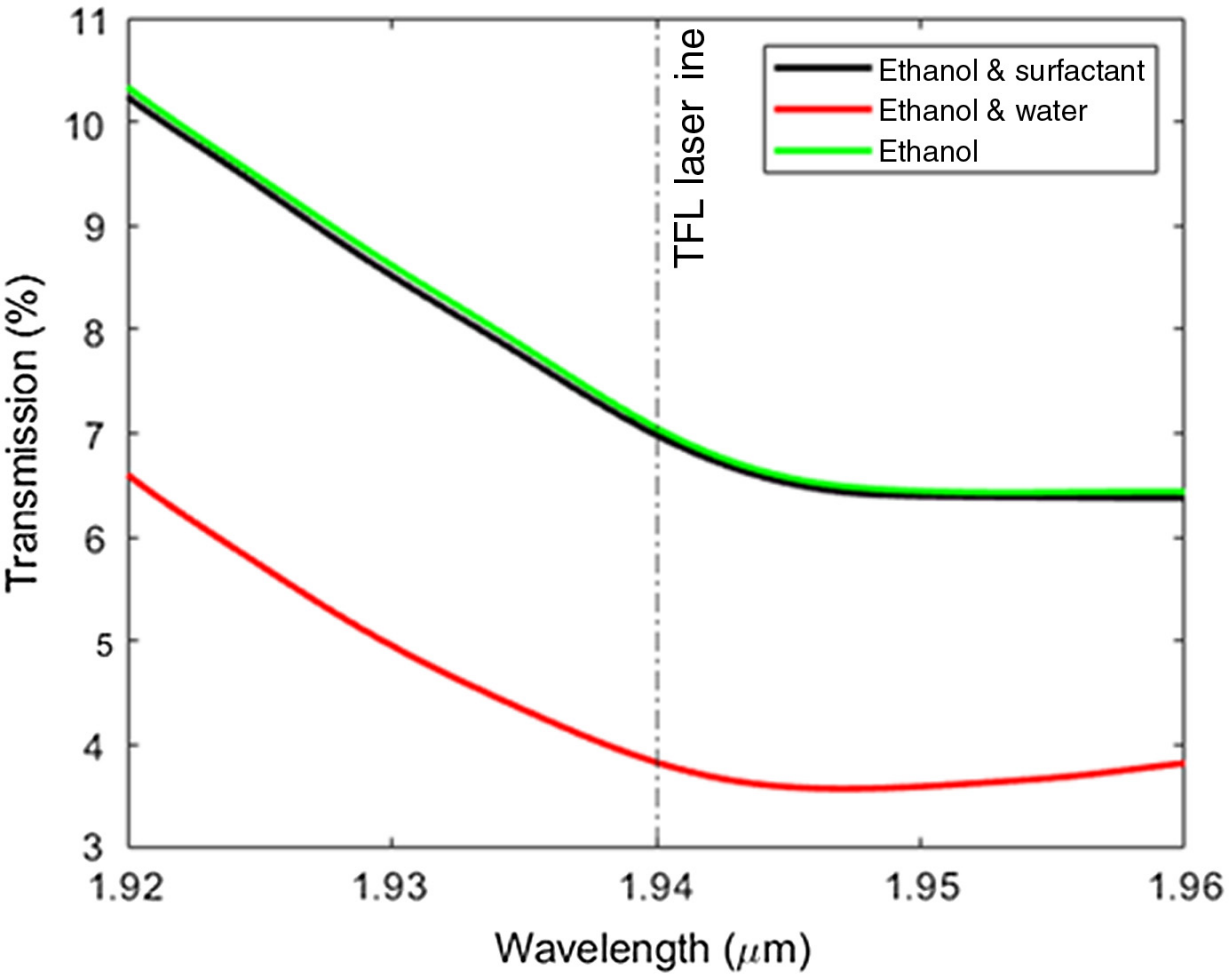
Optical transmission near 1940 nm for 1-cm ethanol 200 proof (in green) and 25  μl surfactant/2500  μl ethanol concentration (in black) shows that the surfactant does not change the optical absorption. However, a 2500  μl ethanol/50  μl water concentration shows a change in optical absorption, demonstrating that water is the primary absorber in the diluted surfactant.

When IR lasers deliver energy through a fiber within a water environment (e.g., the urinary tract accompanied by constant saline irrigation through the working channel of the ureteroscope during lithotripsy), a laser-induced vapor bubble is produced at the distal fiber optic tip. The vapor bubble forms due to high water absorption at these IR wavelengths. A solution containing surfactant was demonstrated to not alter the IR absorption properties and to still produce a vapor bubble with water as the primary absorber (Fig. 1).

This vapor bubble has advantages and disadvantages for the specific application of laser lithotripsy. First, the water present at the distal fiber optic tip is vaporized by the laser energy and in effect “parts the waters” (referred to as the Moses effect^8^^,^^9^), thus enabling subsequent laser pulses to be efficiently transmitted through the vapor channel with very low absorption, to the stone surface for ablation. As a result, IR laser lithotripsy can be conducted in either contact mode or non-contact mode, with the distal fiber tip typically within a few millimeters of the stone surface.

Second, rapid formation of the vapor bubble on a microsecond time scale may create significant pressure transients that are capable of removing some stone material, thus providing a secondary mechanical contribution to ablation,^3^^,^^4^^,^^10^ in addition to the primary photothermal mechanism of long-pulse laser lithotripsy.^11^^,^^12^ These pressures may also contribute to stone “retropulsion,” a unique phenomenon in the field of laser medicine where the target, the stone, moves freely and is propelled backward away from the fiber tip. Stone retropulsion is undesirable in some clinical situations because it may require the surgeon to chase after a moving stone into a region of the urinary tract that is more difficult to access, such as the lower pole of the kidney.

The physics of IR laser-induced vapor bubble formation for biomedical applications has already been studied and reported on in great detail over the past 30 years and is therefore not the main topic of this current study.^11^^–^^24^ Instead, the primary purpose of this preliminary feasibility study is to determine whether a biocompatible surfactant can be mixed at several low concentrations with the normal water irrigation (currently used and typically delivered through the working channel of the ureteroscope) to favorably manipulate the laser-induced vapor bubbles.

## 2 Methods

A TFL (TLR-50/500, IPG Medical, Marlborough, Massachusetts) was used with a center wavelength of 1940 nm. The TFL was operated with pulse energies of 0.05, 0.1, 0.2, and 0.5 J, and pulse durations of 250, 500, 1000, and 2500  μs, providing a constant peak power of 200 W for each set of data. Laser energy was delivered through a 200-μm-core, low-OH, silica optical fiber (BFL22-200, Thorlabs, Newton, New Jersey), with numerical aperture = 0.22 (similar to clinical optical fibers used in lithotripsy). The TFL was modulated by a function generator (DS345, Stanford Research Systems, Sunnyvale, California) to produce a single pulse. A temporal beam profile providing the energy distribution during a single laser pulse was acquired using a photovoltaic IR photodetector (PD-10.6, Boston Electronics, Brookline, Massachusetts) to confirm laser stability. For simplicity and clarity in the graphs, the laser temporal beam profile data were smoothed by a standard Gaussian filter with 10  μs width using MATLAB (Version 2018b, MathWorks, Natick, Massachusetts).

**Fig. 2:**
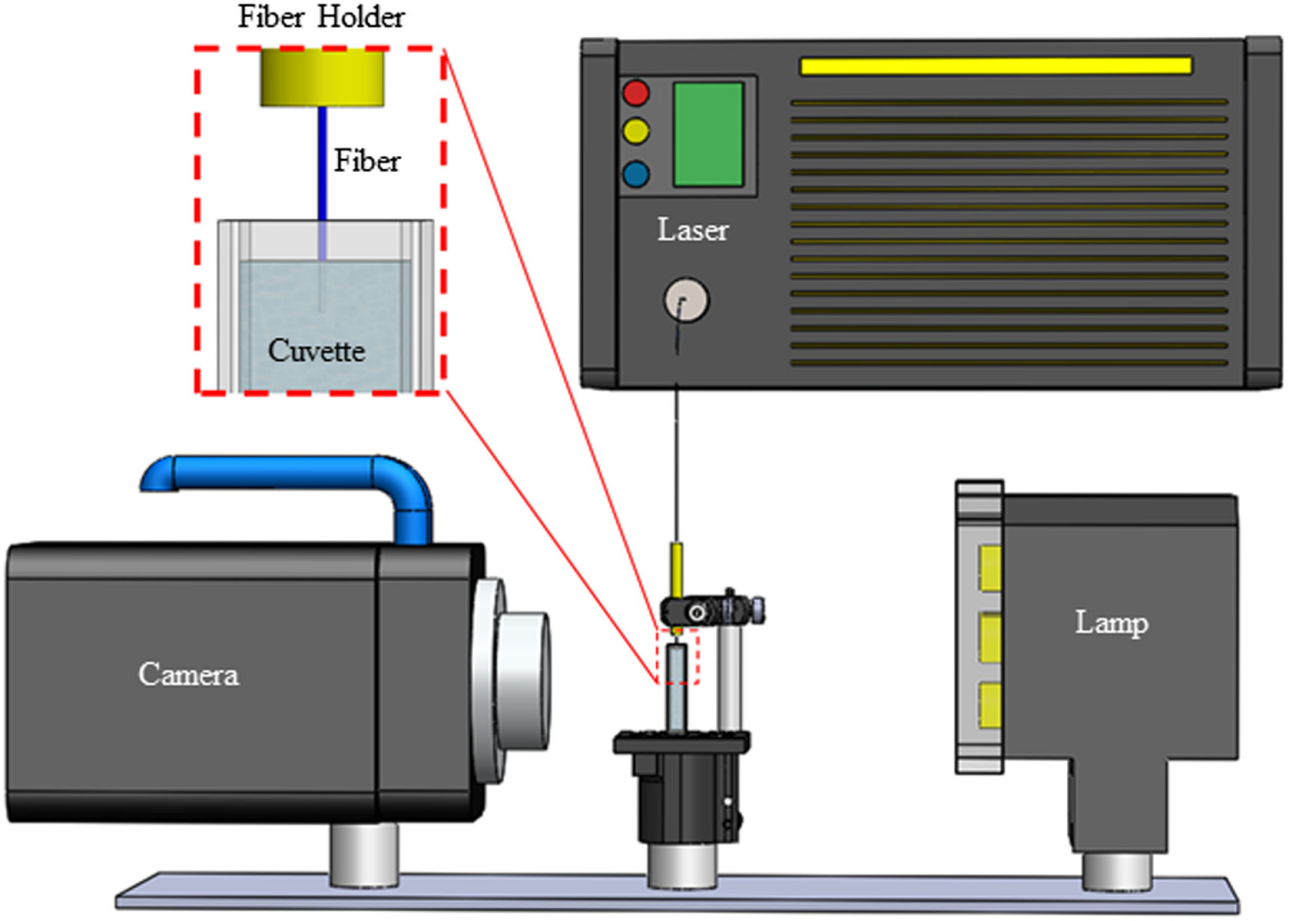
Experimental setup for high-speed imaging of laser-induced vapor bubbles. A magnified view of fiber holder, fiber tip, and cuvette is also shown.

A high-speed camera (Nova S12, Photron, Tokyo, Japan) captured the vapor bubble dynamics. The camera was linked to the laser trigger, enabling capture of a single laser pulse at 200,000 frames per second with a resolution of 256×128  pixels. The optical fiber was polished, clamped, and then submerged into a 4.5-ml (1×1 ×4.5 cm) transparent cuvette. A high-power, light-emitting diode source (Zaila, Nila, Pasadena, California) coupled with an optical diffuser was used as a back light to provide contrast between the fiber optic tip suspended in the solution and the bubbles produced by a single laser pulse (Fig. 2).

**Fig. 3:**
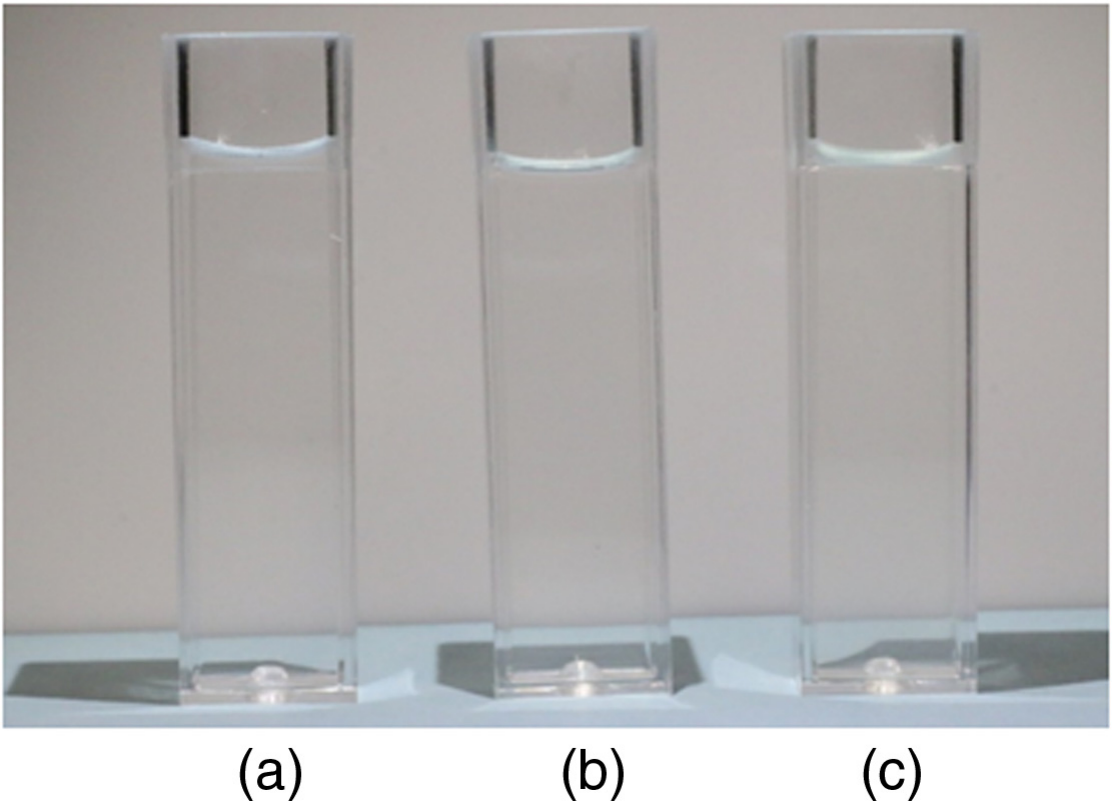
Images showing transparency of cuvettes with (a) deionized water, 0% surfactant, (b) 5% surfactant, and (c) 10% surfactant.

Polysorbate 80, polyoxyethylene sorbitan monooleate (Tween80 Biocompatible Surfactant, Cospheric, Santa Barbara, California) was used as the surfactant for all experiments. The agent was diluted in deionized water to concentrations up to 10%. These concentrations only affected the physical properties of the fluid, but not its physical appearance, which allowed the solution to remain visibly clear (Fig. 3).

To visualize the change in surface tension from 0%, 5%, and 10% surfactant concentrations, a drop of 50  μl of each fluid concentration was pipetted onto a clean glass slide cover. The droplet of each solution concentration was then imaged and the contact angle that each of the droplets made with the glass slide was measured. The measurements were performed using ImageJ software (National Institutes of Health, Bethesda, Maryland). Since surface tension is proportional to the inverse cosine of the contact angle, a smaller contact angle is indicative of a lower surface tension.

**Fig. 4:**
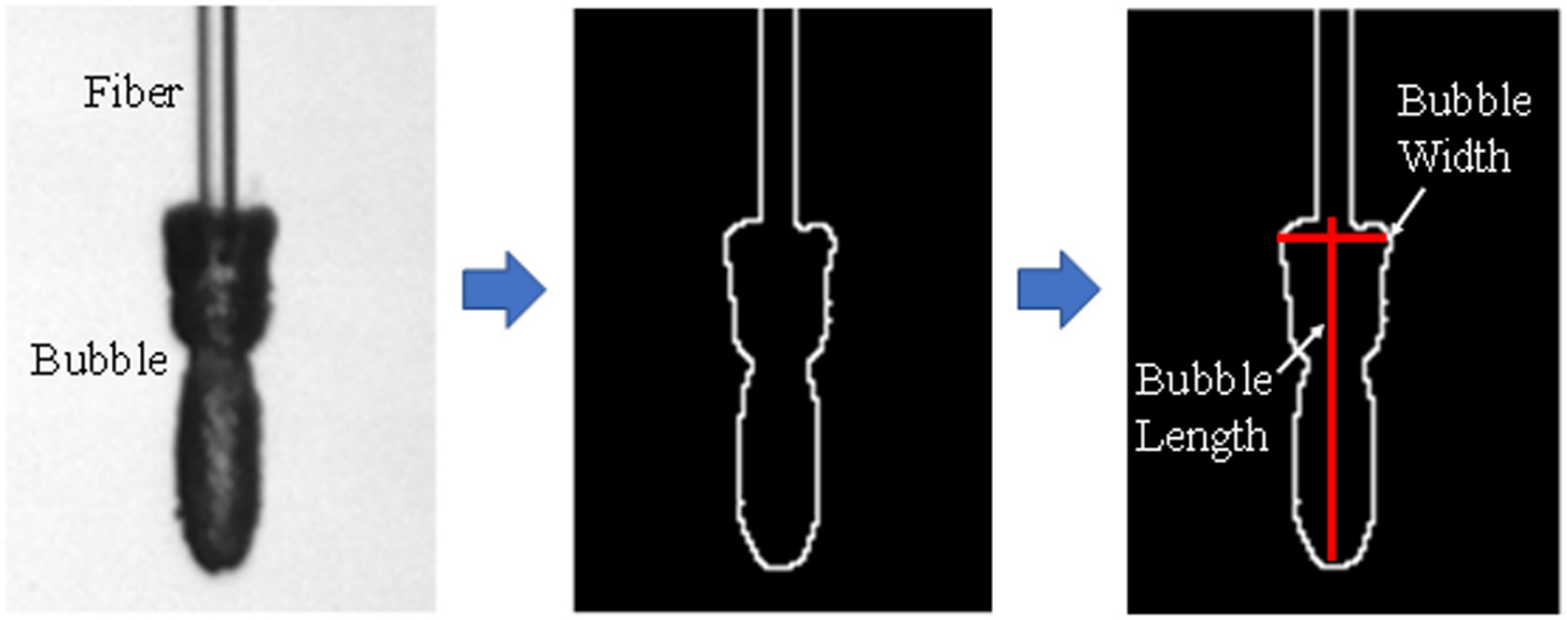
Laser-induced vapor bubble formation was measured using binary analysis and computation of pixel size dimensions with conversion to micrometers. Bubble length (vertical red line) was defined as distance from distal fiber optic tip to bottom of bubble. Bubble width was measured at a location one pixel away from the distal fiber optic tip.

Bubble image data were exported and analyzed in MATLAB. The MATLAB code incorporated edge detection image processing, which first distinguished fiber edges from the background and then detected edges of the bubble. The software tracked bubble expansion while the bubble remained attached to the fiber tip. The code was used to analyze bubble dimensions over time and identified edges of bubble width and length (Fig. 4). Since the exact fiber optic dimensions were known, the distal fiber tip was used as a reference to convert pixel count into micrometers. Two criteria had to be satisfied to record the maximum bubble values for length, width, and duration. First, the bubble needed to remain open because the primary clinical purpose of the first bubble during laser lithotripsy is to provide a low-absorbing vapor channel to the stone surface. Second, the bubble needed to remain attached to the distal fiber optic tip, again for the purpose of providing a continuous channel between the fiber and stone in a clinical procedure. Once the vapor bubbles were detached from the distal fiber optic tip, they were no longer analyzed in this study, which was indicated by the sharp drop in bubble length in the plotted data.

**Fig. 5:**
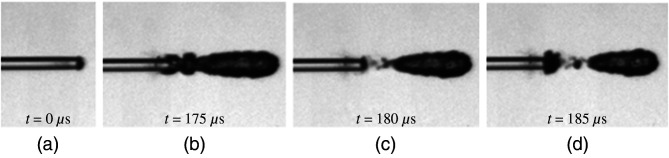
Camera frames showing stages of vapor bubble formation. (a) Formation of new bubble; (b) bubble expanding outward to full size; (c) bubble detaching from fiber optic tip and formation of new bubble; (d) new bubble expanding and original bubble moving away from fiber optic tip.

The code also identified the maximum duration and total number of bubble expansions and collapses for a single laser pulse. The maximum duration was defined as the time duration that a single bubble would expand before it detached from the fiber tip and started its collapse. The total number of bubble expansions and collapses was defined as the total amount of bubble formations during a single laser pulse. The formation of a “new bubble” was defined by where the previous bubble detached itself from the fiber tip (Fig. 5). After batch data were obtained, bubble frames were matched to the data to validate the analysis code.

Statistical analysis of the bubble data (length, width, and duration) was conducted with comparison of the 5% and 10% surfactant data to the control data (water only, 0% concentration) for each of the laser parameter sets tested (n=4 each). A two-tailed t-test was used, with values of p<0.05 considered to be statistically significant, as noted by an asterisk in the tabulated data sets.

The measurements of each of the bubbles was performed without a kidney stone present in order to provide reproducible results. However, a qualitative study was also performed to visualize a bubble interaction with a kidney stone. A 3-mm-diameter calcium oxalate monohydrate (COM) stone was placed at varying distances (1, 2, and 3 mm) from the distal fiber tip. Images of the laser-induced bubble and COM stone interaction were then captured with the high-speed camera experimental setup using a single laser pulse, for 0%, 5%, and 10% surfactant concentrations.

## 3 Results

To confirm that the surfactant concentrations produced a lower surface tension than water, contact angles were measured to be: $\theta_{0\%}\;(35\;\deg)>\theta_{5\%}\;(26\;\deg)>\theta_{10\%}(24\;\deg)\;$ for the 0%, 5%, and 10% surfactant concentrations, respectively.

Table 1 summarizes the data (length, width, and duration) from bubbles formed at 0%, 5%, and 10% surfactant concentrations (n=4). For each individual set of laser parameters (pulse duration in microseconds and pulse energy in Joules) and surfactant concentration (%), the bubble dimensions and lifetime were recorded (average±standard deviation) and observed to be relatively consistent.

**Table 1:** Laser-induced vapor bubble dimensions with percent concentration (of surfactant in solution), average number of bubbles (formed in a single laser pulse), average length (of bubble from fiber tip), average width (of bubble at fiber tip), average duration (lifetime of single bubble during laser pulse), and sample size of n=4 each.

Laser settings	Concentration (%)	Number of bubbles	Length (μm)	Width (μm)	Duration (μs)
250 μs 0.05 J	0	2±0.0	2585±131	1368±77	126±46
	5	1±0.0	3198±46*	1790±28*	214±5*
	10	1±0.0	2585±29	1530±17*	230±11*
500 μs 0.1 J	0	4±0.0	2318±83	1241±60	127±38
	5	2.5±0.8	2875±285*	1437±135	239±61*
	10	1±0.0	2695±62*	1464±25*	532±13*
1000 μs 0.2 J	0	6.5±0.5	2350±185	1319±85	151±19
	5	5±0.5	2733±225*	1588±220	217±99
	10	3.3±0.4	3028±315*	1473±58*	309±90*
2500 μs 0.5 J	0	13.3±3.0	2430±91	1311±76	186±114
	5	6.5±1.7	3743±405*	1563±89*	352±177
	10	5.3±1.6	4185±535*	1411±18	528±423

* Denotes statistically significant difference (p<0.05) for bubble length, width, or duration, compared to control study (water, 0% concentration).

**Fig. 6:**
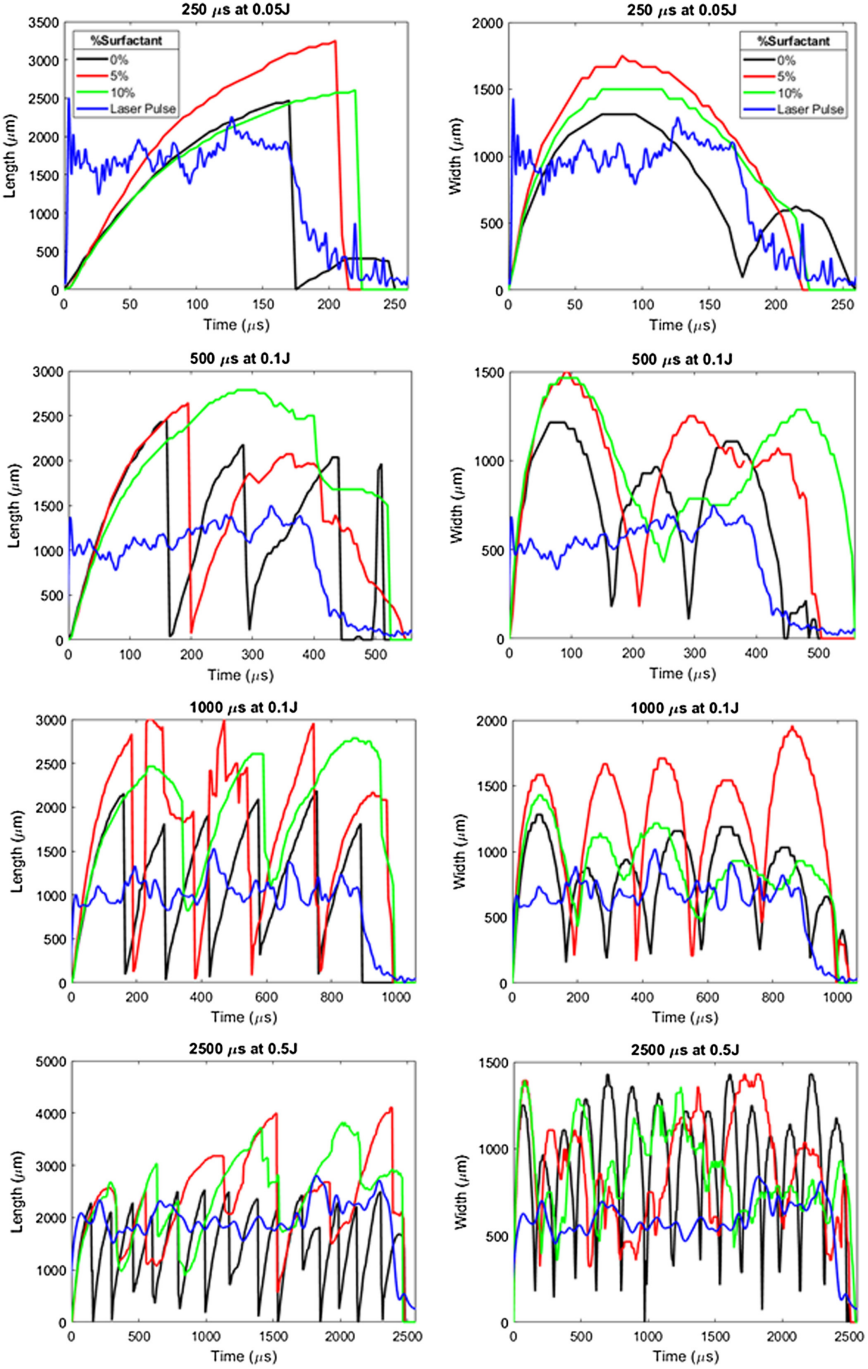
TFL-induced vapor bubble dimensions while bubbles remained attached to the fiber tip, as a function of time and surfactant concentration (black: 0%, red: 5%, and green: 10%), superimposed with averaged laser pulse profile (blue).

Figure 6 plots laser pulse energies of 0.05, 0.1, 0.2, and 0.5 J and pulse durations of 250, 500, 1000, and 2500  μs, for surfactant concentrations of 0% (in black), 5% (in red), and 10% (in green). The temporal beam profile for each laser pulse is also shown (in blue). As the laser pulse begins, the bubble starts to expand, and as the laser pulse continues, the bubble continues its expansion until it detaches from the fiber tip and collapses. At this time point, a second bubble forms and the original bubble begins to collapse. As the laser pulse ends, the bubble stops expanding and eventually collapses. The 5% and 10% concentrations increased vapor bubble width, length, and duration versus the control study (water) without surfactant (0%). Bubble expansion occurred at similar rates; however, bubble formation in low surface tension solutions lasted longer, which in turn resulted in longer lasting and larger volume bubbles.

Table 1 and Fig. 6 also show the total number of bubbles formed during a single laser pulse. Individual bubbles are represented by distinct peaks in the data. As an example, for the specific laser parameters of 500  μs and 0.1 J, when observing both bubble length and width, there are an average of four peaks when laser irradiation occurs in water, compared with an average of 2.5 peaks for a 5% surfactant concentration, and only one peak for a 10% concentration. This trend of fewer bubble formations for 5% and 10% concentrations is also observed within all of the other sets of laser parameters as well (250  μs at 0.05 J, 1000  μs at 0.2 J, and 2500  μs at 0.5 J). Fewer bubbles formed in viscous solutions for all concentrations tested (5% and 10%), compared to water (0%). Bubbles formed in 5% solution were about 29% longer, 22% wider, and 72% longer lasting, whereas bubbles formed in 10% solution were 29% longer, 12% wider, and 169% longer lasting than bubbles formed in water (Table 1). The asterisks in the individual data sets denote a statistically significant result (p<0.05) between the individual bubble characteristics (length, width, or duration) for 5% and 10% surfactant concentrations versus the control study (water, 0%).

**Fig. 7:**
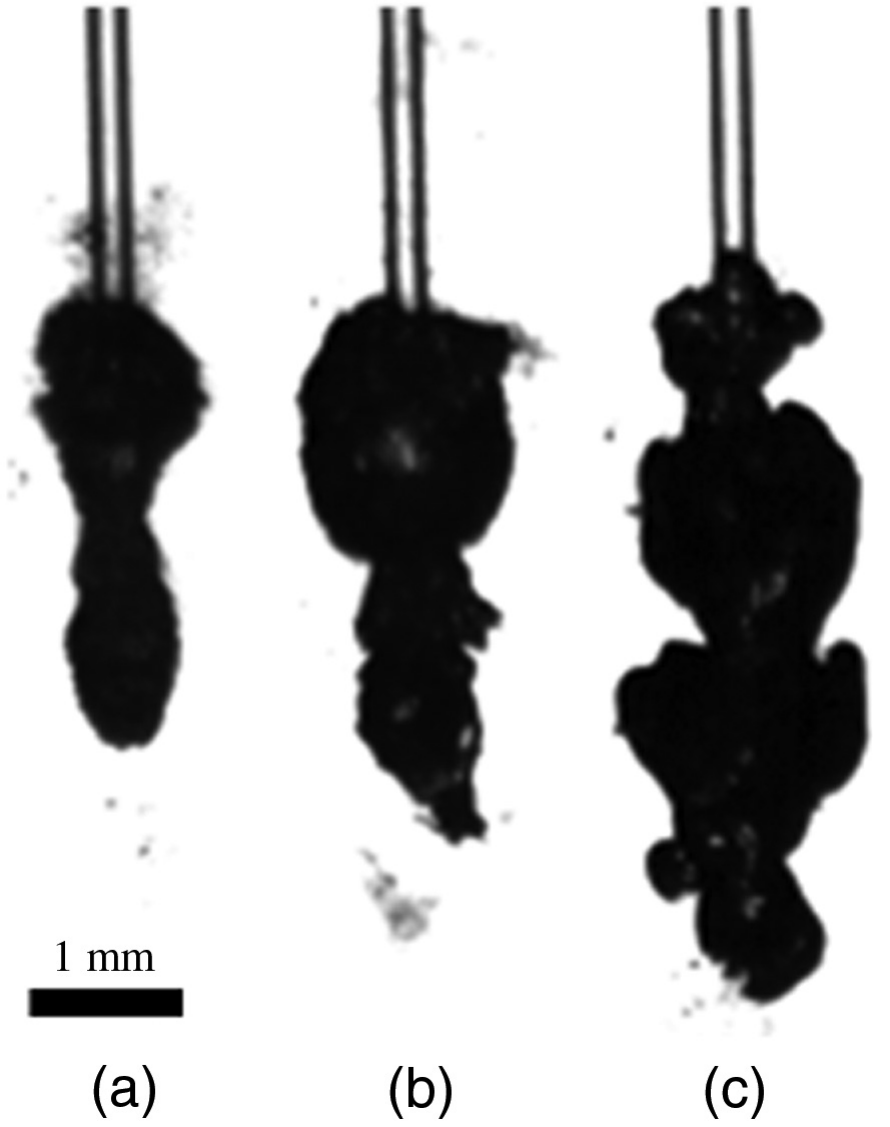
Photographs of laser-induced vapor bubbles (2500  μs and 0.5 J) at peak length dimensions: (a) water, control; (b) surfactant (5%); and (c) surfactant (10%). Images were taken at the same scale.

**Fig. 8:**
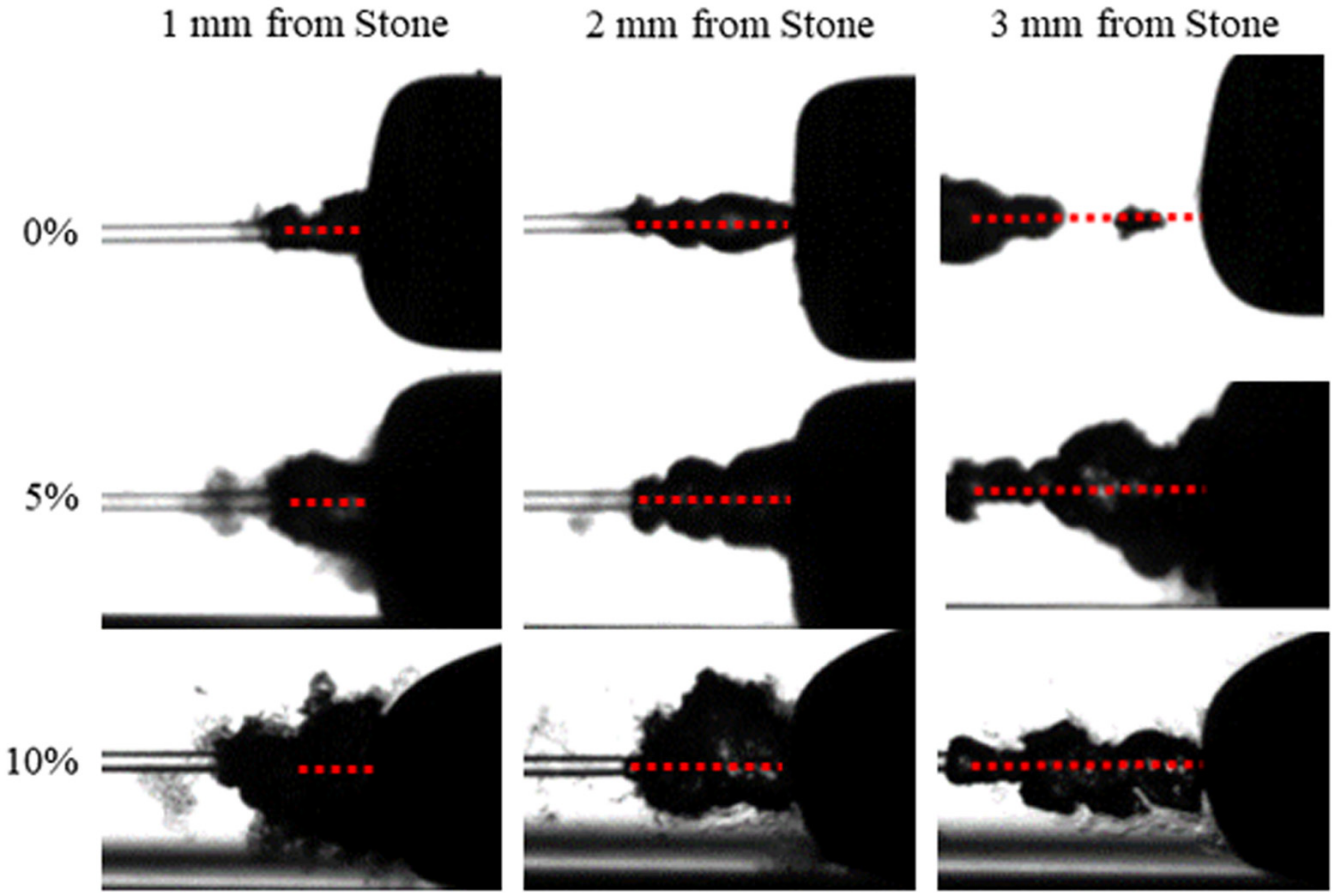
Photographs of laser-induced vapor bubbles near a COM kidney stone, at varying working distances from the fiber optic tip of 1, 2, and 3 mm. The red dashed lines show the distance between the distal fiber tip and stone surface.

If surfactant was present in the solution, then the bubble length increased. This was also true for studies performed when a COM kidney stone was present. Figure 7 shows a single laser pulse of 2500  μs at 0.5 J at maximum length for different surfactant concentrations. The bubble formations for a single laser pulse were captured with the high-speed camera at variable fiber tip to stone working distances of 1, 2, and 3 mm (Fig. 8).

## 4 Discussion

During long-pulse IR laser lithotripsy, laser energy exiting from the fiber tip is highly absorbed by the water, resulting in a vapor bubble. This bubble provides a low-absorbing vapor channel for laser energy to reach the kidney stone, even in non-contact mode, at a short distance of a few millimeters. The vapor bubble also produces strong pressure transients, resulting in a mechanical contribution to stone ablation, as well as stone movement (retropulsion).^24^

This study, using a biocompatible surfactant to manipulate laser-induced vapor bubble dimensions and durations, was motivated by several factors. First, either a lower laser energy may be used to create a bubble of similar dimensions or an equivalent laser energy may be used to create a larger vapor bubble, thus potentially translating into a more efficient laser lithotripsy process.

Second, a “popcorn” laser lithotripsy technique is frequently utilized to treat urinary stones located in hard-to-reach locations, such as the calyces of the kidney. In these situations, removal of stone fragments with a stone basket device is not practical due in part to the sharp ureteroscope flexion angles required and the number of stones present. Instead, laser energy is intentionally delivered into the fluid medium, resulting in fluid jetting and turbulent flow upon collapse of the vapor bubbles. The objective is to enable periodic movement of the stones in close proximity with the fiber optic tip for gradual fragmentation into smaller pieces. Use of a surfactant to produce larger vapor bubble dimensions may potentially contribute to making this popcorn ablation method more efficient as well.

Third, larger vapor bubble lengths may enable laser lithotripsy to be performed at greater non-contact working distances between the distal fiber optic tip and the stone sample so that precise positioning of the fiber relative to the stone is less critical to efficient stone ablation.

The results of this preliminary study demonstrated that TFL irradiation in viscous solutions utilizing a biocompatible and commercially available surfactant created larger volume and longer duration vapor bubbles. The specific surfactant used in these studies (Tween-80) was chosen based on multiple factors, including its ability to lower the surface tension of a solution, its biocompatibility, its commercial availability, and its potential as an irrigation substitute during laser lithotripsy. This surfactant remained clear at low concentrations, which is an important feature of irrigation since it maintains a clear field of view for the surgeon during laser lithotripsy.

It should be noted that previous initial experiments were performed with lower surfactant concentrations of only 1% to 5% and limited to low sample sizes.^25^ This current study therefore focused on the use of higher concentrations (up to 10%) and higher sample sizes (n=4) for each set of individual laser parameters, with comparison of water to both 5% and 10% surfactant concentrations.

The underlying basic principle for bubble formation involves the pressure differential between the inside, the outside, and the surface tension of the bubble. Bubbles formed in pure water are inherently unstable due to their high surface tension, $7.2\times 10^{-2}\;N/m$ at 25°C, resulting in rapid bubble collapse. However, the addition of a surfactant into water at low concentrations lowers the surface tension of the bubble, in effect making the bubble more stable. To satisfy these conditions, and for the bubble to be more stable, the pressure of the gas vapor inside the bubble, $P_{\mathrm{in}}$, has to be larger than the outside bubble pressure plus pressure created by surface tension:^26^

$$P_{\text{in}}=P_{\text{out}}+2s/r,$$

where $P_{\text{out}}$ is all of the atmospheric pressure and water pressure pushing inward on the newly forming bubble, s is the surface tension of solution that the bubble is forming in, and r is the radius of the bubble. The addition of Tween-80 surfactant to water at a concentration of only 1% lowers the surface tension to $3.8\times 10^{-2}\;N/m$.^27^ Lower surface tension translates into formation of larger volume bubbles, longer duration bubbles, and a fewer number of bubbles.

Since 5% and 10% surfactant concentrations produced similar bubble dimensions (length and width), the pressure of the solution in the cuvette might hinder the formation of a larger bubble at surfactant concentrations greater than 5%. The 1 cm width of the cuvette may also have possibly adversely affected the bubble measurements at higher surfactant concentrations. However, it was observed that the bubble duration correlated with surfactant concentration. This may be explained by higher viscosity solutions taking a longer time for bubbles to collapse. It is also possible that when consecutive bubbles form during a single laser pulse, the transient pressure of the initial bubble collapse may negatively impact the formation of the following bubbles. This may explain, in part, some of the statistically insignificant results and high standard deviations shown for the longer pulse duration data in Table 1. Overall, there is a significant difference between individual bubble characteristics (length, width, and duration) for the 5% and 10% surfactant concentrations, as compared to the control study (water, or 0% surfactant) in the majority of studies.

As summarized in Table 1, the maximum lengths and widths of the bubbles are on average about 29% longer and 17% wider when produced inside viscous solutions, with concentrations from 5% and 10%. This increase in maximum bubble dimensions (length and width) for bubbles attached to the fiber tip directly translated into a longer channel that the laser energy can efficiently traverse within a low absorbing vapor medium. The effects of a larger volume bubble during TFL ablation of a kidney stone are observed as a longer working distance, as shown in Fig. 8. The surfactant-enhanced bubbles increase the effective working distance for non-contact stone ablation. A larger volume bubble forming near the stone is also observed when surfactant is used. It is speculated that the collapse of a larger volume bubble may help facilitate fragmentation of the kidney stone in a shorter total irradiation time.

Table 1 also shows that during a single laser pulse, fewer bubbles are formed for higher surfactant concentrations. Fewer bubbles connected to the fiber tip for a longer duration also effectively indicates an extended channel, thus allowing more laser energy to propagate without absorption by water. A longer bubble duration also equates to fewer bubble collapsing events, which may also potentially limit stone retropulsion effects.

During future experiments, pressure transients created by bubble collapse in surfactant will need to be quantified and compared with bubbles formed in a water medium. If pressure transients are observed to be larger in surfactant solutions, then greater amounts of stone material may be removed due to stronger mechanical contributions to ablation. However, if the pressures created in a surfactant solution are excessive, bubble collapse and fluid jets may also contribute to stone retropulsion. This phenomenon of stone movement may potentially be desirable for creating turbulent flow during the popcorn technique of laser lithotripsy within the confined spaces of the kidney but otherwise may be undesirable for treating stones in the ureter that are not impacted and are therefore free to move away from the fiber and into more difficult to reach locations in the urinary tract, such as the calyces of the kidney.

Finally, it should be briefly noted that there is significant interest within the lithotripsy field in multiple different novel techniques that enable manipulation of bubbles near the stone surface. For example, several recent reports have shown that microbubbles can be custom designed to have an affinity for urinary stones. The bubbles are attracted to the stone surface, where the bubbles can then be manipulated and exploded using extracorporeal ultrasonic methods.^28^^–^^30^

## 5 Conclusions

The addition of a commercially available, biocompatible surfactant to water at concentrations up to 10% produces laser-induced vapor bubble dimensions with 29% greater lengths, 17% greater widths, and 120% longer bubble durations than water alone. Although further study is warranted, surfactant-enhanced vapor bubbles may potentially result in more efficient laser lithotripsy through the use of lower pulse energies and/or greater stone ablation rates, longer effective non-contact working distances between the fiber and stone, and may also play a role in enhancing the popcorn technique currently used during treatment of stones located in the kidney.
